# Quality Variation of Pork Bellies by Cutting Manner and Quality Grade

**DOI:** 10.3390/foods13193129

**Published:** 2024-09-30

**Authors:** Pil-Nam Seong, Jeong-Ah Lee, Dong-Heon Song, Hyun-Wook Kim, Dong-Gun Kim, Samooel Jung, Van-Ba Hoa

**Affiliations:** 1 Animal Products Utilization Division, National Institute of Animal Science, Rural Development Administration (RDA), Wanju 55365, Republic of Korea; spn202@rda.go.kr (P.-N.S.); j2970703@rda.go.kr (J.-A.L.); sdh8507@rda.go.kr (D.-H.S.); woogi78@rda.go.kr (H.-W.K.); kdg6589@rda.go.kr (D.-G.K.); 2 Department of Animal Science and Biotechnology, Chungnam National University, Daejeon 34134, Republic of Korea; jso@rda.go.kr

**Keywords:** pork belly, meat quality, fatty acid, aroma

## Abstract

In the meat industry, the quality grading system is commonly applied to classify carcasses based on quality and value. Presently, to facilitate consumer convenience, pork bellies are prepared into slices and retailed in supermarkets and butchers. The objective of this study was to assess the effect of quality grade (QG) and retail cutting manner on the quality properties of pork bellies. Thirty-two bellies with different QGs: QG1^+^, 1, 2, and off-grade (*n* = 8 each) randomly collected from a commercial slaughterhouse were used. Each belly was cut into 3 portions: A (5–10th rib, cranial edge), B (11–15th rib), and C (without rib, caudal edge) according to the commonly used retail cutting manner. Samples were subjected to chemical composition, quality traits, and aroma analysis. Fat content was highest in QG1^+^ and lowest in off-grade and distributed at a higher level in portions A and B than in portion C in all QGs (*p* < 0.05). Off-grade was associated with higher shear force and chewiness values and lower levels of palmitic and stearic acids, regardless of the cutting portion. The bellies used in this study exhibited variations in chemical composition and quality properties not only among the QGs but also across the cutting portions within each QG.

## 1. Introduction

Pork belly is a valuable part, accounting for about 12% of the total live weight of each pig [1]. In many countries, belly cut is highly preferred by consumers due to its delicious taste, despite its higher price compared to the rest of the pork carcass [2,3,4,5]. Depending on the geographical areas, belly cut is used for different purposes, such as producing bacon or making grilled dishes [3,4,6]. Meat quality is a complex concept influenced by many factors; for instance, the chemical composition (e.g., fat and moisture contents) affects the color and water-holding capacity and eating quality (e.g., flavor) of the meat [7]. In the meat industry, grading standards systems have been developed and used to classify carcasses based on quality and value. This grading system helps quality-based payment to meat producers and is a motivational basis for the producers to provide markets with high-quality and value products through improving the production environment, such as feeding regimes [8]. According to Korean standards, pork carcasses are classified into 4 categories: QG1^+^, 1, 2, and off-grade, thus influencing the wholesale or retail price. For example, according to wholesale prices in 2021, each kilogram of cold carcass costs about 5307, 5211, 5170, and 2919 Korean Won for the QG1^+^, 1, 2, and off-grade, respectively [8]. It is well known that the belly is composed of multiple layers of lean and fat tissues. The quality and distribution of fat layers play an important role in affecting consumer acceptability and technological and nutritional values [9]. Presently, for the consumer’s convenience, pork belly is often cut into slices with different sizes for retail at butcheries and supermarkets, with most consumers prefer the belly slices with more layers of lean–fat intertwined with each other. As a result, too much fat or lean cuts are often less preferred by the consumer, and they must reduce their selling prices. However, even though they belong to the same QG group, the quantity and distribution of lean–fat layers differ depending on the anatomical location and physiological function. Previous studies have shown a wide variation in meat quality between sampling locations within each belly [10,11]. In our previous studies, we have found that the QG has a significant impact on the chemical composition, fat content, and overall pork quality [12].

Assessing the influence of anatomical location or cutting manner on quality property is necessary for determining the reasonable prices for different portions within each belly in the same QG and is the basis for standardization of cutting manner of the pork bellies by consumer preferences (more fat or lean favor) in different markets. Until now, only a few studies have examined the impact of anatomical locations (cutting manner) and quality grade on the pork belly’s quality, as mentioned earlier. However, no studies have been conducted to assess the impact of both cutting manner and quality grade on the quality of pork belly. Thus, the objective of this study was to evaluate the impacts of QG and retail cutting manner on the physicochemical, quality and flavor properties of pork belly.

## 2. Materials and Methods

### 2.1. Sample Preparation

**Figure 1:**
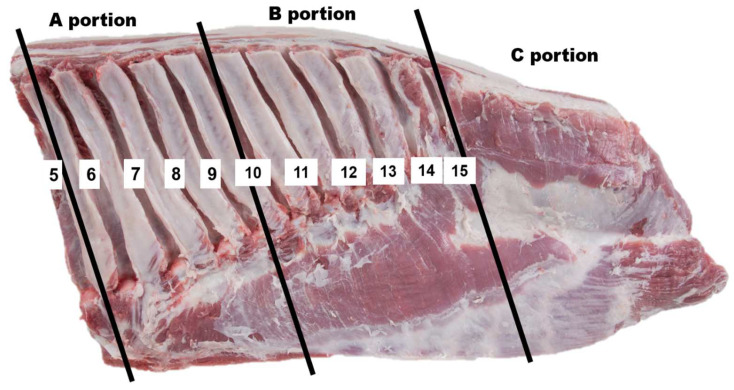
Preparation of portions: (A) (cranial edge: 5–10th rib), (B) (middle, 11–15th rib), and (C) (remaining caudal edge) of bellies.

Fresh bellies (in the form of primal cut) of growing-finishing crossbred pigs ([Landrace × Yorkshire] ♀ × Duroc ♂) (LYD) were collected from a commercial slaughterhouse (Jeonju, Republic of Korea) after 24 h of slaughter. Before collection, the pork carcasses were evaluated for quality grades (QG) according to the Korean pork carcass grading system by the Korea Institute for Animal Products Quality Evaluation [13], detailed in our previous study [7]. For instance, QG1^+^ included pigs with hot carcass weight (83–93 kg), back-fat thickness (17–25 mm), good marbling (measured on the cross-sectional surface of loin at the 5–6th vertebrae after 30 min blooming), meat color values of 3–5 and fat color values of 2–3. QG1: pigs with hot carcass weight of 80–83 kg, back-fat thickness (15–28 mm), fine marbling, meat color values of 3–5, and fat color values of 1–3; QG2: pigs with hot carcass weight of 98–109 kg, back-fat thickness (14–28 mm), poor marbling, meat color values of 2 and 6, and fat color values of 4–5; Off-grade: all remaining carcasses not in the QG1^+^, 1 and 2 above. Finally, 32 carcasses (16 castrated males and 16 females, *n* = 8 per QG) were chosen, and the belly samples from the left sides of carcasses were collected, vacuum-packaged, and immediately transported to the Laboratory of Meat Science (National Institute of Animal Science, Wanju-gun, Republic of Korea) with a transport time of about 30 min. Upon arrival, the belly samples were immediately used for quality analysis. Similar to the cutting manner used for retail sale in supermarkets or butchers, each belly was cut into three different portions: A (cranial edge: 5–10th rib), B (middle, 11–15th rib), and C (remaining caudal edge) as demonstrated in Figure 1. From these portions, they were further divided into 2.5 cm thick slices for analysis of meat quality properties. Furthermore, in each analysis, the sampling location was also fixed for all the portions in all the QGs.

### 2.2. Meat Quality Analysis

Crude fat, protein, moisture, and collagen contents were determined with a Food Scan (Lab 78810, Foss Tecator Co., Ltd., Hillerod, Denmark) as described by Anderson [14]. Briefly, the meat was blended using a meat grinder (Hanil Co., Chungcheongnam-do, Republic of Korea), and then approximately 100 g of each sample was placed in a petri dish, spread evenly, and introduced into the device. Each sample was analyzed in duplicate.

The pH values of meat were measured in triplicate using a pH meter (Mettler Toledo GmbH, Greifensee, Switzerland) after homogenizing the samples (3.0 g each) with 27 mL distilled water at 11,000 rpm for 30 s.

Water-holding capacity (WHC) was determined using the method of Fischer et al. [15] with minor modifications. Briefly, after the chemical composition was completed, an exact 1.00 g of each sample was weighed, placed in a 10 mL tube, and centrifuged at 70,000× *g* for 30 min at 10 °C using an Avanti JXN-26 centrifuge (Beckman Coulter, Fullerton, CA, USA). After that, a piece of Whatman filter paper (No. 2) was inserted into the bottom of the sample tube and left for 1 h at 4 °C to absorb the water released after the centrifugation. The weights of the Whatman filter paper before and after absorbing the released water were weighed to determine the WHC value. As follows:

$$\mathrm{WHC}(\%)=\mathrm{Moisture}-\left\{\frac{W1-W2}{W3}\times 100\right\}$$

W1, W2: the weight (gram) of Whatman filter paper after and before absorbing the released water. W3: the weight (g) of the sample.

Meat and fat color: The values of color traits (CIE L*, a* and b*) were measured on the cross-sectional surfaces of the samples after 30 min blooming using CR-400 colorimeter (Minolta Camera Co., Osaka, Japan) with a D65 illuminant, 2 °C observer and 8 mm measuring aperture. Before use, the color meter was calibrated with a standard white tile (Y = 86.31, X = 0.31, and Y = 0.32). The meat or fat color values were obtained after measuring at five random areas of each sample, avoiding measuring in areas with fat (for measuring the meat color) or lean (for measuring the fat color). The color measurement was carried out in a cooling room (4 °C).

Cooking loss and shear force were measured on the same slices (2.5 cm thick slices) of each sample after cooking the slices at 180 °C using an electrical grill (Tefal Corp., Rumilly, France) until the internal temperature reached 75 °C. The weights of the samples before and after cooking were weighed to determine the cooking loss. After that, five strips (2 cm in length) per slice were prepared using a 0.5-inch metal corer. The shear force values (kg/cm^2^) were obtained after completely cutting the sample strips using a V-shaped shear blade attached to a Universal Testing Machine (Model 5543, Instron Corp., High Wycombe, UK) at 200 mm/min crosshead speed and load cell of 40 N.

### 2.3. Fatty Acid Profiles

To extract lipids, meat samples (10 g each) were homogenized with 40 mL of chloroform: diethyl ether (1:1 ratio) mixture solution for 3 min at 2500 rpm, according to the protocol of Folch et al. [16]. Before homogenizing, 1 mL of triundecanoin (5 mg/mL, as an internal standard, Sigma-Aldrich, Saint Louis, MI, USA) was also added to the sample. After the homogenates were filtered through No. 2 Whatman filter paper, about 20 g of Na_2_SO_4_ was added to the filtrate to absorb water. After that, the samples were concentrated at 55 °C under vacuum, and the resultant concentrated lipid was hydrolyzed with 1 mL of 0.5 N NaOH and 1 mL tricosanoic acid at 100 °C for 15 min in a heating block. After cooling at room temperature for 10 min, the fatty acid methyl esters (FAMEs) were prepared by adding 2 mL of 14% BF3-methanol at 100 °C for 30 min in a heating block. Finally, 1 mL of heptane was added to each of the samples and vortexed for 1 min. The upper phase containing FAMEs (about 0.5 mL) was carefully collected and placed into a test vial for analysis. The FAMEs were analyzed with a gas chromatograph (GC) equipped with a flame ionization detector (Agilent, 8890, Bruker, Bremen, Germany). For separation of the fatty acids, a 1.0 μL sample was injected into a column (100 m × 0.25 mm × 0.2 μm; SP-2560, Supelco, Bellefonte, PA, USA), and nitrogen was used as a carrier gas at a constant flow rate of 0.8 mL/min. The initial GC oven, injection, and detection temperatures were 80, 225, and 285 °C, respectively. For the identification of fatty acids, reference standards (F.A.M.E. Mix C4-C24, Supelco) were separated under the same condition. Each fatty acid was identified by comparing its retention time with that of the known standard. The concentration of fatty acids was calculated using the known amount of internal standard and expressed as g/100 g meat.

### 2.4. Aroma Component Analysis

Aroma compounds generated in the meat during cooking were analyzed using our previously developed method [17]. First, the meat samples were cooked on a frying pan at 180 °C for approximately 2 min. After that, an aliquot (1.0 g) of each sample was immediately taken, placed into a 20 mL vial, and closed tightly to avoid the loss of volatile aromas. We used the solid phase micro-extraction technique to extract the aromas from the cooked samples, and this process was performed using an automated robotic system (model: PAL RSI 85) connected to a gas chromatography (GC, model: 8890)-mass spectrometry (MS, 5977B MS, Agilent Technologies, Santa Clara, CA, USA). For the extraction, we inserted a 75-μm carboxen–polydimethylsiloxane fiber (Supelco) into the vial and kept it at 60 °C with a shaking speed of 60 rpm for 50 min. Next, the fiber was inserted into the GC at the injection port at 250 °C for 5 min. Separation of aromas was performed using a column (30 m × 0.25 mm i.d. × 0.25 μm film thickness; Agilent, Folcom, CA, USA) and helium as a carrier gas. The analysis was then carried out with the GC/MS system under conditions similar to those described by Hoa et al. [18]. We used the retention time of external standards separated under similar conditions to identify the aromas. The concentration of each compound was calculated using a known amount of internal standard (1.0 μL of 2-methyl-3-heptanone, 816 mg/mL, added to the sample vial before subjecting it to the extraction process). Finally, we classified the aromas into separate groups (e.g., aldehydes, alcohols, nitrogen and sulfur compounds, etc.) based on their chemical properties.

### 2.5. Statistical Analysis

Data were analyzed using the SAS package (SAS Institute, Cary, NC, USA). Two-way analysis of variance (ANOVA) was performed in which quality grade and cutting manner were the fixed factors, and the obtained data was set as variables. The results were expressed as mean ± standard error. The means comparison was carried out using Duncan’s multiple-range test. Significance was set at the level of 0.05.

## 3. Results and Discussion

### 3.1. Effect on Chemical Composition

The fat, protein, moisture, and collagen content in the bellies by retail cutting manner and QG are presented in Table 1. Regarding the protein, we observed a large variation depending on the portions within each QG, with the highest being 17.12% and the lowest being 12.87%. The portion C had the highest protein level compared to the remaining portions (A and B), regardless of QG (*p* < 0.05). Fat is an important component affecting the overall technological and eating quality aspects of meat of almost all species [11,18]. Especially for pork belly, the quality and quantity of fat dramatically affect the melting point, which consequently affects processing properties and the final yield of processed products (e.g., bacon) [19]. Our results showed that cutting manner and QG significantly affected the fat level: In portions A and B, the off-grade had about 10% lower fat content than the other remaining QGs (1^+^, 1, and 2). Meanwhile, in portion C, the off-grade had about 3–5% lower fat content than those in the remaining QGs. For the cutting manner, portion A had the highest fat content (ranging from 31 to 41%), followed by portion B (26–40%), and the lowest in portion C (20–25%), regardless of QG (*p* < 0.05). Hoa et al. [7] reported an average fat level (19–32%) in bellies of the same pig breed. In line with our study, Albano-Gaglio et al. [11] found a large variation in fat content (24–61%) in the bellies of Iberian × Duroc crossbred pigs. Similar to our cutting manner, Soladoye et al. [20] found a higher fat level in the cranial portion than in the caudal portion of bellies. In contrast to our cutting manner, Trusell et al. [21] prepared pork bellies into dorsal, central, and ventral portions, and these authors reported the highest fat level (over 60%) in the dorsal portion, followed by central and ventral (31%) portions. Similarly, Albano et al. [22] reported a fat content of about 37% in the central section of bellies, and this level was almost similar to that found in portion B in our study.

According to results of consumer survey studies conducted by Vonada et al. [23] and Lee and Kim [3], most Korean consumers prefer pork belly with moderate fat level (around 30%), whereas bellies with fat content lower than 14% or higher than 42% is less preferred. Regarding moisture content, its level was also significantly affected by the cutting manner and QG. In contrast to the fat content, the highest moisture content was found in portion C compared to the other portions (A and B) in all the QGs (*p* < 0.05). This is consistent with the trend observed in previous studies [11,12]. From our results and those reported in previous studies as mentioned above, it may be said that the protein, fat and moisture contents are highly variable depending on the cutting portions, even within the same QG. This may be due to the distinctive anatomical location and physiological function (e.g., muscle contraction level), leading to different levels of fat and protein accumulation in each portion studied [24].

### 3.2. Effect on Meat Quality Properties

The effects of QG and cutting manner on the meat quality are presented in Table 2. The meat pH was significantly influenced by the QG: higher pH values were found in QG1^+^ than in the QG 2 or off-grade (*p* < 0.05). However, no effects of the cutting manner on meat pH were observed in all the QGs except for the off-grade (*p* > 005). pH is an important factor directly affecting the overall quality of meat. After slaughter, the glycolytic process under anaerobic conditions is the main mechanism leading to the decline in meat pH [25]. Thus, the difference in the lean ratio (protein content, Table 1) affecting the stored glycogen source may be the leading cause of the difference in meat pH values among the GQs. In agreement with our results, previous studies have also reported that pH values are generally higher in belly portions with higher fat content [10].

Until now, numerous methods have been developed to measure the WHC of meat [26]. In this study, we used both cooking and centrifugal methods. However, no effects of the cutting manner or QG on the WHC were observed (*p* > 0.05). Tenderness is a very important factor in eating quality, satisfaction, and purchasing decisions for almost all types of meat consumers [27]. Shear force is an important indicative parameter reflecting the tenderness of meat. We observed that both the cutting manner and QG significantly affected the shear force values of meat. Among all the QGs, portion A had the lowest value, whereas portion C had the highest value (*p* < 0.05). Many factors, such as chemical composition, especially fat content, have been found to directly affect the tenderness of meat. Researchers have found that meat with higher fat content generally has lower shear force values and vice versa [27,28]. This is because the presence of fat cells reduces the protein density and weakens the connective tissue, leading to a decrease in the shear force values of the meat after cooking [29]. We assume that, in this study, the difference in fat levels may be the main reason underlying the difference in shear force values of meat among the portions (A, B, and C) and QGs. Similarly, previous studies have also found that pork with higher fat content has lower shear force values [30].

### 3.3. Effect on Color of Lean and Fat

Color traits of lean and fat layers measured on the cross-sectional surface of bellies are shown in Table 3.

For the lean layer, QG and cutting manner generally had a negligible effect on the L* (lightness) and a* (redness). The impact of the QG was only observed in portion C, where the off-grade had a higher L* value than QG2 (*p* < 0.05). Meanwhile, the cutting manner only affected the redness in QG1^+^, with a higher value in portion C than in portion A (*p* < 0.05). For yellowness (b*), it was influenced both by the QG (off-grade had a higher value than QG1 and 1^+^) and cutting manner (portion C had a lower value than the portion A or B in most QGs (*p* < 0.05). Regarding the fat color, the lightness varied among the QGs: the off-grade had significantly lower L* values than the remaining QGs at most portions. However, the QG did not affect the a* and b* values (*p* > 0.05). In agreement with our results, Knecht et al. [10] found a significant difference in L*, a*, and b* values of lean meat and fat among cutting locations of pork bellies. Consumer’s perception of a meat product is primarily based on its color, which determines whether they will purchase the meat or not [31]. The color of meat is affected mainly by anatomical locations or muscle types and different fat:lean ratios [10]. Furthermore, light penetration differs depending on the degree of fatty acid saturation in different layers of fat [32]. According to results obtained from survey studies on consumer preference for pork color over the years in different countries, it has been shown that 54% of surveyed French consumers choose the dark color, 72% of surveyed Taiwanese consumers choose the dark-red color, 56% of surveyed Canadian consumers choose the light-red color, and 31% of surveyed Mexico consumers choose the dark color [33,34,35,36]. Based on color measurement results, portion C in off-grade was lighter in color than in the remaining QGs. Meanwhile, in QG1^+^, portions B and C were redder in color than portion A.

### 3.4. Effect on Fatty Acid Profiles

The outcome of the analysis shows that there was a wide variation in fatty acid profiles among the portions and QGs (Table 4). Among 11 saturated fatty acids (SFA) found in all the samples, palmitic acid (C16:0) had the highest concentration (7.38–12.51 g/100 g), followed by stearic acid (C18:0; 3.40–6.27 g/100 g), while the remaining fatty acids exhibited negligible amounts. The highest amounts of C16:0 and C18:0 were found in the QG1^+^, 1, and 2 compared to the off-grade, regardless of the cutting portion (*p* < 0.05). In agreement with our results, Marcos et al. [37] found that pork bellies with a high-fat level contain a higher palmitic content. In all the QGs, however, the cutting manner did not affect the C16:0 content. This implies that the accumulation of this fatty acid is similar across the cutting portions and is only influenced by the quality grade. Oleic acid (C18:1n9) was the most predominant monounsaturated fatty acid (MUFA), with levels ranging from 13.72 g/100 g to 22.17 g/100 g depending on QGs and cutting portions. Except for portion B, the QG did not affect the oleic acid content in all portions studied. Meanwhile, the cutting manner significantly (*p* < 0.05) affected C18:1n9 level in all QGs except QG1^+^. For linoleic acid (C18:2n6), the effect of the QG was observed only in portions A and B, while the impact of the cutting manner was observed in all QGs except QG1^+^. Similar to our results, Albano-Gaglio et al. [11] show that C16:0 and C18:0 contents tended to increase, whereas C18:2n6 content tended to decrease in belly portions with higher fat levels. The remaining longer-chain fatty acids (C20:5n3, C21:0, C22:0, C22:1n9, C24:0, and C24:1n9) were found at relatively low concentrations and were unaffected by the cutting manner or QG (*p* > 0.05).

The fatty acid composition indicates nutritional value and plays a vital role in meat’s texture and aromatic characteristics (e.g., desirable or undesirable flavor) after cooking [20,38]. For monogastric animals, such as pigs, fatty acids in meat are generated from dietary fatty acids (adipogenesis) and de novo biosynthesis [39]. Fatty acids often accumulate in fat depots with different proportions depending on animal age, breed, gender, and anatomical location [18,24,40]. Compared to the concentrations of particular fatty acids in bellies in this study, considerably higher contents of C18:1n9, C18:2n6, and C18:3n3 have been reported for another cut (*Longissimus dorsi*) of other pig breeds in the literature [41,42]. From the point of view of health effects, intake of SFAs (e.g., C16:0) enriched diets are often linked to a high risk of increased serum cholesterol levels and cardiovascular disease [43]. Additionally, diets rich in MUFAs, such as C18:1n9 and n-3 polyunsaturated fatty acids (PUFAs), often exhibit beneficial effects, such as reducing cardiovascular diseases and improving physiological functions, while diets rich in n-6 fatty acids may cause adverse health effects, such as inflammatory and allergic disorders [44,45].

### 3.5. Effect on Aroma Compounds

The aroma of meat, created by volatiles and sensed by the smell buds in the nose, is the essential sensory attribute determining the overall eating quality of cooked meat [46]. After cooking (e.g., steaming, frying, or roasting), the cooked meat often bears a characteristic aroma as a result of multiple chemical reactions, such as the Maillard reaction, thermal degradation of fatty acids, and the interaction between intermediates derived from these two reactions [47]. The aroma of cooked meat is influenced by many factors, such as cooking conditions; however, under the same cooking conditions, the more important factor affecting the aroma is the chemical composition (e.g., fat and protein contents) of meat [48]. According to analysis results using the SPME-GC/MS system, 18 aldehydes, 6 alcohols, 4 sulfur and nitrogen compounds, and 7 hydrocarbons were identified and quantified. The total amount (summed up from all identified compounds) of each aroma class was calculated, statistically analyzed, and compared among the cutting portions and QGs (Table 5). Regarding the aldehyde class, the highest content was found in QG1^+^, followed by QG1, 2, and off-grade, regardless of cutting portions (*p* < 0.05). The cutting manner did not affect the total aldehyde content in QG1^+^ and QG1, but it did affect the QG2 and off-grade with a higher amount in portion A than in portion B or C (*p* < 0.05). In both portions A and B, the total alcohol content was higher in QG1^+^ and QG1 than in other remaining QGs (*p* < 0.05). The cutting manner did not affect the alcohol content in all QGs except the off-grade. Regarding the hydrocarbon content, no effect of QG was found, but the cutting manner significantly affected this content in almost all QGs. It is well known that thermal oxidation of fatty acids, mainly unsaturated fatty acids (e.g., C18:1n9 and C18:2n6), is the main pathway for producing the aldehydes, alcohols, and hydrocarbons [38,49]. Alcohols and hydrocarbons appear to have a negligible role in developing cooked meat aroma because they have a high odor detection threshold. However, aldehydes (e.g., octanal, nonanal, decanal) play a significant role in the development of aromas of cooked meat because they usually possess pleasant odors (e.g., fatty and sweet) and a low odor detection threshold [47]. Previous studies have shown that amounts of aldehydes produced in cooked meat increase with increasing fat content [50,51]. We also assume that the difference in fat content may be the main reason for the variations in the concentrations of aldehyde, alcohol, and hydrocarbon classes in cooked meat among the cutting portions and QGs.

The sulfur and nitrogen-containing compounds are mainly produced in the Maillard reaction by reducing sugars and amino acids, and they often possess pleasant odors, such as meaty and roasty, etc., even in small amounts [47]. Interestingly, this aroma class was not found in portion A of almost all QGs, which had higher fat contents (Table 1). This could be partly because high concentrations of fat-derived compounds (e.g., aldehydes) might limit their formation or reduce their concentrations. This phenomenon has also been stated in previous studies [49,50,51]. Lastly, the total amount of all classes of aromas was found to be the highest in all cutting portions of the QG1^+^ (2.3–2.6 µg/g), followed by QG1, QG2, and off-grade (1.05–1.42 µg/g) (*p* < 0.05). Meanwhile, the cutting manner did not affect the total amount of aroma classes in all QGs except off-grade. From the results of aroma analysis in this study, it may be said that all portions of QG1^+^ with a higher total aldehydes content may be associated with a stronger fatty odor note, while portions B and C of QG1, QG2, and off-grade with the presence of sulfur and nitrogen compounds may be associated with a meaty or roasted odor note.

## 4. Conclusions

Based on the results obtained from this study, it may be concluded that there was a wide variation in chemical composition (e.g., fat, protein, fatty acids, and aroma compounds) and quality (e.g., shear force, pH, color.) among the cutting portions within each the quality grade. This study provides consumers with useful information, therefore helping them to choose the preferred portions of the belly. Additionally, based on the results of this study, meat processors and retailers can develop other cutting manners that are more suitable for consumer categories (e.g., preference for more fat or lean) in different markets, as well as give appropriate prices for the corresponding cutting portions within each belly of each QG. On the other hand, it is also suggested that a quality grading system capable of classifying pork bellies with more uniform quality across all cutting portions should be developed, through which the quality-based payment (quality grade-based payment) to producers will be more accurate.

## Figures and Tables

**Table 1:** Proximate composition of belly as affected by the retail cutting and quality grade.

Traits	Retail Cut	Quality Grade			
		1^+^	1	2	Off-Grade
Protein (%)	A	12.89 ± 1.27 ^Bc^	13.25 ± 0.88 ^Bc^	12.87 ± 1.76 ^Bb^	14.60 ± 1.94 ^Ac^
	B	14.30 ± 1.54 ^Bb^	14.16 ± 1.56 ^BCb^	13.10 ± 1.94 ^Cb^	15.77 ± 2.33 ^Ab^
	C	16.64 ± 0.84 ^Ba^	16.44 ± 1.03 ^Ba^	16.33 ± 1.60 ^Ba^	17.12 ± 1.61 ^ABa^
Fat (%)	A	41.50 ± 6.60 ^Aa^	39.96 ± 5.40 ^Aa^	40.37 ± 8.48 ^Aa^	31.22 ± 10.90 ^Ba^
	B	35.90 ± 8.62 ^Ab^	36.27 ± 6.40 ^Ab^	40.14 ± 9.38 ^Aa^	26.97 ± 11.76 ^Ba^
	C	25.76 ± 5.02 ^Ac^	21.37 ± 5.61 ^Bc^	23.21 ± 5.15 ^Bb^	20.70 ± 8.22 ^Bb^
Moisture (%)	A	45.72 ± 5.46 ^Bc^	47.13 ± 4.50 ^Bc^	46.81 ± 6.47 ^Bb^	53.85 ± 8.58 ^Ab^
	B	49.71 ± 6.92 ^Bb^	49.58 ± 4.82 ^Bb^	46.39 ± 7.56 ^Bb^	56.84 ± 9.15 ^Ab^
	C	57.01 ± 4.93 ^Ca^	60.23 ± 2.77 ^ABa^	57.94 ± 5.44 ^BCa^	61.62 ± 6.43 ^Aa^
Collagen (%)	A	1.93 ± 0.15	1.93 ± 0.17	1.67 ± 0.53	1.94 ± 0.16
	B	2.02 ± 0.30	2.09 ± 0.14	1.73 ± 0.63	2.00 ± 0.23
	C	2.00 ± 0.24	1.85 ± 0.19	1.67 ± 0.68	1.93 ± 0.23

^A–C^ Means with different superscripts in the same row significantly differ at *p* < 0.05. ^a–c^ Means with different superscripts in the same column significantly differ at *p* < 0.05.

**Table 2:** Meat quality properties of belly as affected by the retail cutting and quality grade.

Traits	Retail Cut	Quality Grade			
		1^+^	1	2	Off-Grade
Cooking loss (%)	A	31.51 ± 3.37	31.60 ± 3.13	32.06 ± 2.33	30.78 ± 3.7
	B	29.17 ± 2.76	29.32 ± 4.63	29.58 ± 3.08	30.01 ± 4.42
	C	27.67 ± 4.44	30.98 ± 3.82	29.45 ± 5.51	29.62 ± 4.25
Water-holding capacity (%)	A	73.85 ± 6.33	70.07 ± 5.64	70.13 ± 3.77	69.92 ± 12.36
	B	73.01 ± 7.01	72.41 ± 8.05	66.24 ± 6.25	71.07 ± 11.77
	C	77.41 ± 7.22	74.95 ± 4.86	73.03 ± 6.27	73.92 ± 11.17
Shear force (kg/cm^2^)	A	2.48 ± 0.65 ^Bb^	2.66 ± 0.73 ^ABc^	2.56 ± 0.54 ^Bb^	2.79 ± 0.54 ^Ac^
	B	2.30 ± 0.76 ^Cb^	3.01 ± 0.63 ^ABb^	2.77 ± 1.12 ^Bb^	3.21 ± 0.98 ^Ab^
	C	3.24 ± 1.03 ^Ba^	3.67 ± 0.62 ^Aa^	3.46 ± 0.58 ^ABa^	3.51 ± 0.64 ^Aa^
pH	A	6.20 ± 0.26 ^A^	6.05 ± 0.24 ^AB^	5.98 ± 0.28 ^B^	5.96 ± 0.30 ^Ba^
	B	6.04 ± 0.19 ^A^	5.90 ± 0.28 ^AB^	5.90 ± 0.34 ^AB^	5.77 ± 0.23 ^Bb^
	C	6.03 ± 0.26 ^A^	5.85 ± 0.25 ^AB^	5.81 ± 0.32 ^B^	5.70 ± 0.21 ^Bb^

^A–C^ Means with different superscript in the same row significantly differ at *p* < 0.05. ^a–c^ Means with different superscript in the same column significantly differ at *p* < 0.05.

**Table 3:** Meat and fat color of belly as affected by the retail cutting and quality grade.

Traits		Retail Cut	Quality Grade			
			1^+^	1	2	Off-Grade
Meat	CIE L*	A	50.91 ± 2.65	52.10 ± 4.05	51.54 ± 3.72	52.42 ± 4.01
		B	51.37 ± 4.80	52.26 ± 4.33	51.40 ± 3.21	53.24 ± 3.47
		C	50.09 ± 6.51 ^AB^	50.66 ± 5.60 ^AB^	49.75 ± 6.71 ^B^	52.96 ± 6.31 ^A^
	CIE a*	A	11.43 ± 1.97 ^b^	12.55 ± 2.35	12.54 ± 2.10	12.18 ± 2.32
		B	12.50 ± 2.04 ^a^	12.66 ± 1.52	13.12 ± 2.09	12.85 ± 2.21
		C	12.63 ± 2.31 ^a^	12.93 ± 3.97	11.92 ± 3.00	13.11 ± 3.39
	CIE b*	A	6.06 ± 1.24 ^B^	6.60 ± 1.09 ^ABab^	6.85 ± 1.43 ^Aa^	6.91 ± 1.35 ^Aa^
		B	6.15 ± 1.18 ^B^	7.01 ± 1.79 ^Aa^	6.67 ± 1.34 ^ABa^	6.82 ± 1.27 ^ABa^
		C	5.93 ± 1.52 ^AB^	5.94 ± 1.87 ^ABb^	5.37 ± 1.30 ^Bb^	6.17 ± 1.50 ^Ab^
Fat	CIE L*	A	79.98 ± 1.56 ^Ab^	80.93 ± 2.74 ^A^	80.06 ± 2.01 ^Ab^	78.54 ± 2.16 ^B^
		B	80.19 ± 2.37 ^b^	81.27 ± 1.69	80.22 ± 2.34 ^ab^	80.41 ± 2.15
		C	81.15 ± 1.83 ^Aa^	81.39 ± 2.47 ^A^	81.18 ± 1.68 ^Aa^	79.79 ± 3.35 ^B^
	CIE a*	A	3.31 ± 0.96 ^b^	3.03 ± 1.92	3.44 ± 1.41	3.23 ± 1.44
		B	4.00 ± 0.95 ^a^	3.07 ± 1.12	3.93 ± 1.70	3.80 ± 1.14
		C	3.83 ± 1.11 ^a^	3.63 ± 1.19	3.20 ± 1.53	3.62 ± 1.58
	CIE b*	A	7.32 ± 0.98 ^b^	6.88 ± 1.60	6.80 ± 1.55	6.73 ± 1.73
		B	7.96 ± 1.03 ^a^	7.11 ± 1.31	7.51 ± 1.94	7.64 ± 1.61
		C	7.79 ± 1.25 ^ab^	7.70 ± 1.29	6.98 ± 1.39	7.00 ± 1.87

^A,B^ Means with different superscript in the same row significantly differ at *p* < 0.05. ^a,b^ Means with different superscript in the same column significantly differ at *p* < 0.05.

**Table 4:** Fatty acid composition (g/100 g) of belly as affected by the retail cutting and quality grade.

Items	Retail Cut	Quality Grade			
		1^+^	1	2	Off-Grade
C10:0 (Capric acid)	A	0.06 ± 0.01 ^aA^	0.05 ± 0.01 ^aA^	0.05 ± 0.01 ^aA^	0.04 ± 0.00 ^aB^
	B	0.03 ± 0.00 ^bC^	0.05 ± 0.01 ^aA^	0.04 ± 0.01 ^bB^	0.03 ± 0.00 ^bC^
	C	0.05 ± 0.01 ^aA^	0.02 ± 0.02 ^bC^	0.03 ± 0.00 ^cBC^	0.04 ± 0.01 ^aAB^
C12:0 (Lauric acid)	A	0.09 ± 0.01 ^aA^	0.06 ± 0.01 ^bB^	0.05 ± 0.01 ^bB^	0.04 ± 0.01 ^bC^
	B	0.05 ± 0.01 ^bA^	0.07 ± 0.00 ^aA^	0.07 ± 0.01 ^aA^	0.04 ± 0.01 ^bB^
	C	0.05 ± 0.01 ^b^	0.05 ± 0.01 ^b^	0.04 ± 0.00 ^c^	0.05 ± 0.01 ^a^
C14:0 (Myristic acid)	A	0.72 ± 0.13 ^AB^	0.78 ± 0.10 ^A^	0.76 ± 0.09 ^aA^	0.58 ± 0.10 ^bB^
	B	0.71 ± 0.11 ^A^	0.73 ± 0.06 ^A^	0.84 ± 0.13 ^aA^	0.51 ± 0.10 ^bB^
	C	0.80 ± 0.18 ^A^	0.67 ± 0.17 ^AB^	0.59 ± 0.07 ^bB^	0.76 ± 0.12 ^aAB^
C14:1 (Myristoleic acid)	A	0.02 ± 0.01	0.03 ± 0.00 ^A^	0.03 ± 0.00	0.02 ± 0.00
	B	0.04 ± 0.01	0.03 ± 0.00	0.03 ± 0.00	0.02 ± 0.01
	C	0.03 ± 0.01	0.03 ± 0.01	0.03 ± 0.00	0.04 ± 0.01
C15:0 (Pentadecanoic acid)	A	0.03 ± 0.01 ^b^	0.04 ± 0.00	0.03 ± 0.01 ^b^	0.04 ± 0.01
	B	0.06 ± 0.01 ^aA^	0.03 ± 0.00 ^B^	0.03 ± 0.00 ^bB^	0.03 ± 0.01 ^B^
	C	0.04 ± 0.01 ^b^	0.04 ± 0.01	0.06 ± 0.01 ^a^	0.04 ± 0.01
C16:0 (Palmitic acid)	A	10.85 ± 2.01 ^A^	11.17 ± 1.71 ^A^	11.02 ± 1.43 ^A^	8.42 ± 1.40 ^B^
	B	10.38 ± 1.80 ^A^	9.29 ± 0.95 ^A^	12.51 ± 2.10 ^A^	7.38 ± 1.31 ^B^
	C	11.41 ± 2.31 ^A^	9.67 ± 2.33 ^AB^	11.60 ± 1.99 ^A^	8.27 ± 1.14 ^B^
C16:1 (Palmitoleic acid)	A	0.94 ± 0.18 ^AB^	1.12 ± 0.15 ^A^	1.08 ± 0.15 ^aA^	0.86 ± 0.16 ^B^
	B	0.81 ± 0.58 ^B^	1.02 ± 0.19 ^aAB^	1.26 ± 0.21 ^aA^	ND
	C	1.25 ± 0.29 ^A^	ND	0.35 ± 0.50 ^bB^	1.36 ± 0.20 ^A^
C17:0 (Margaric acid)	A	0.14 ± 0.03 ^aB^	0.17 ± 0.02 ^aB^	0.16 ± 0.02 ^B^	0.17 ± 0.05 ^A^
	B	0.20 ± 0.14 ^aA^	0.15 ± 0.02 ^aB^	0.17 ± 0.02 ^AB^	ND
	C	0.01 ± 0.00 ^bB^	0.01 ± 0.00 ^bB^	0.09 ± 0.13 ^AB^	0.16 ± 0.07 ^A^
C17:1 (Heptadecenoic acid)	A	0.11 ± 0.03 ^b^	0.16 ± 0.02 ^ab^	0.13 ± 0.02 ^b^	0.16 ± 0.04 ^ab^
	B	0.20 ± 0.03 ^a^	0.13 ± 0.02 ^b^	0.15 ± 0.02 ^b^	0.13 ± 0.04 ^b^
	C	0.18 ± 0.05 ^a^	0.19 ± 0.05 ^a^	0.20 ± 0.03 ^a^	0.20 ± 0.04 ^a^
C18:0 (Stearic acid)	A	5.75 ± 1.05 ^A^	5.70 ± 0.91 ^aA^	5.81 ± 0.84 ^aA^	4.49 ± 0.64 ^aB^
	B	5.34 ± 1.07 ^A^	4.31 ± 0.53 ^bA^	6.27 ± 1.10 ^aA^	3.99 ± 0.66 ^bB^
	C	5.49 ± 1.02 ^A^	4.78 ± 1.07 ^bAB^	3.94 ± 0.59 ^bB^	3.40 ± 1.00 ^bA^
C18:1 n9 (Oleic acid)	A	17.59 ± 0.37	20.09 ± 0.41 ^a^	19.29 ± 0.42 ^ab^	16.58 ± 0.19 ^ab^
	B	16.52 ± 1.31 ^B^	14.90 ± 1.36 ^bB^	22.17 ± 1.67 ^aA^	13.72 ± 0.18 ^bB^
	C	20.83 ± 1.43	18.53 ± 1.58 ^ab^	15.77 ± 2.17 ^b^	20.96 ± 1.63 ^a^
C18:2 n6 (Linoleic acid)	A	8.29 ± 0.49 ^A^	6.67 ± 0.92 ^bAB^	6.12 ± 0.76 ^bB^	6.34 ± 0.58 ^bB^
	B	7.42 ± 0.43 ^AB^	6.38 ± 0.73 ^bB^	8.44 ± 1.04 ^aA^	6.27 ± 0.84 ^bB^
	C	7.92 ± 0.12	8.32 ± 0.13 ^a^	8.41 ± 0.08 ^a^	6.86 ± 0.37 ^a^
C18:3 n-3 (Linolenic acid)	A	0.36 ± 0.06 ^b^	0.39 ± 0.05	0.36 ± 0.04 ^b^	0.31 ± 0.08 ^b^
	B	0.36 ± 0.07 ^b^	0.38 ± 0.01	0.49 ± 0.06 ^a^	0.29 ± 0.07 ^b^
	C	0.42 ± 0.23 ^aAB^	0.38 ± 0.02 ^B^	0.41 ± 0.01 ^abB^	0.49 ± 0.03 ^aA^
C20:0 (Arachidic acid)	A	0.10 ± 0.02 ^aB^	0.08 ± 0.01 ^B^	0.13 ± 0.02 ^aA^	0.08 ± 0.01 ^B^
	B	0.05 ± 0.04 ^abB^	0.07 ± 0.01 ^B^	0.14 ± 0.07 ^aA^	ND
	C	0.05 ± 0.05 ^b^	ND	0.02 ± 0.03 ^b^	0.08 ± 0.02
C20:1 (Eicocenoic acid)	A	0.30 ± 0.04 ^B^	0.41 ± 0.07 ^aAB^	0.45 ± 0.06 ^aA^	0.41 ± 0.08 ^aAB^
	B	0.38 ± 0.08 ^B^	0.32 ± 0.04 ^bB^	0.54 ± 0.09 ^aA^	0.30 ± 0.09 ^bB^
	C	ND	0.01 ± 0.00 ^c^	0.01 ± 0.01 ^b^	0.01 ± 0.00 ^c^
C20:3 n3 (Eicosatrienoic acid)	A	0.03 ± 0.01	0.05 ± 0.01	0.05 ± 0.01 ^a^	0.05 ± 0.01 ^ab^
	B	0.06 ± 0.01 ^AB^	0.05 ± 0.01 ^BC^	0.07 ± 0.01 ^aA^	0.04 ± 0.02 ^bC^
	C	0.07 ± 0.02 ^A^	ND	0.02 ± 0.03 ^bB^	0.06 ± 0.01 ^aA^
C20:5 n3 (Eicosapentaenoic acid)	A	0.01 ± 0.00	0.01 ± 0.00	0.01 ± 0.00	0.01 ± 0.00
	B	0.01 ± 0.00	0.01 ± 0.00	0.01 ± 0.00	0.01 ± 0.00
	C	0.01 ± 0.00	0.01 ± 0.00	0.01 ± 0.00	0.01 ± 0.00
C21:0 (Heneicocanoic acid)	A	0.01 ± 0.00	0.01 ± 0.00	0.01 ± 0.00	0.02 ± 0.00
	B	0.01 ± 0.00	0.01 ± 0.00	0.01 ± 0.00	0.01 ± 0.00
	C	ND	0.01 ± 0.00	0.01 ± 0.00	0.01 ± 0.00
C22:0 (Behenic acid)	A	0.02 ± 0.00	0.02 ± 0.00	0.02 ± 0.00	0.01 ± 0.01
	B	0.01 ± 0.00	0.02 ± 0.00	0.02 ± 0.00	0.01 ± 0.00
	C	0.01 ± 0.00	0.01 ± 0.00	0.02 ± 0.01	0.01 ± 0.00
C22:1 n9 (Erucic acid)	A	0.01 ± 0.00	0.02 ± 0.00	0.02 ± 0.00 ^b^	0.02 ± 0.00
	B	0.01 ± 0.00	0.02 ± 0.00	0.03 ± 0.00 ^a^	0.01 ± 0.01
	C	0.01 ± 0.00	ND	0.01 ± 0.00 ^c^	0.02 ± 0.00
C24:0 (Lignoceric acid)	A	0.01 ± 0.01	0.01 ± 0.00	0.01 ± 0.00	ND
	B	0.01 ± 0.00	0.01 ± 0.00	0.01 ± 0.00	0.01 ± 0.00
	C	0.01 ± 0.00	0.01 ± 0.00	0.01 ± 0.01	0.01 ± 0.00
C24:1 n9 (Nervonic acid)	A	0.01 ± 0.00	0.01 ± 0.00	0.01 ± 0.00	0.01 ± 0.00
	B	0.01 ± 0.00	0.01 ± 0.00	0.01 ± 0.00	0.01 ± 0.00
	C	0.01 ± 0.00	0.00 ± 0.00	0.00 ± 0.00	0.01 ± 0.00

ND: Not detectable. ^A–C^ Means with different superscript in the same row significantly differ at *p* < 0.05. ^a–c^ Means with different superscript in the same column significantly differ at *p* < 0.05.

**Table 5:** Total amount (µg/g) of class of aroma component in belly as affected by the retail cutting and quality grade.

Aroma Class	Retail Cut	Quality Grade			
		1^+^	1	2	Off-Grade
Ʃ Aldehyde	A	2.21 ± 0.25 ^A^	1.90 ± 0.05 ^B^	1.56 ± 0.13 ^aC^	1.30 ± 0.10 ^aD^
	B	2.15 ± 0.13 ^A^	1.84 ± 0.08 ^B^	1.29 ± 0.09 ^bCD^	1.15 ± 0.04 ^bD^
	C	2.38 ± 0.08 ^A^	1.86 ± 0.06 ^B^	1.26 ± 0.07 ^bC^	0.90 ± 0.03 ^cD^
Ʃ Alcohols	A	0.08 ± 0.01 ^A^	0.07 ± 0.00 ^cB^	0.06 ± 0.00 ^C^	0.07 ± 0.00 ^aB^
	B	0.08 ± 0.01 ^B^	0.08 ± 0.00 ^bB^	0.13 ± 0.06 ^A^	0.07 ± 0.00 ^aB^
	C	0.09 ± 0.01 ^A^	0.09 ± 0.00 ^aA^	0.05 ± 0.00 ^C^	0.06 ± 0.00 ^bB^
Ʃ Hydrocarbons	A	0.05 ± 0.01 ^c^	0.10 ± 0.02 ^ab^	0.04 ± 0.00	0.05 ± 0.00 ^b^
	B	0.07 ± 0.01 ^b^	0.11 ± 0.01 ^a^	0.04 ± 0.00	0.06 ± 0.00 ^a^
	C	0.12 ± 0.01 ^a^	0.08 ± 0.01 ^b^	0.05 ± 0.00	0.06 ± 0.00 ^a^
Ʃ Sulfur and nitrogen compounds	A	ND	ND	ND	0.01 ± 0.00 ^b^
	B	ND	0.01 ± 0.00 ^b^	0.01 ± 0.00	0.01 ± 0.00 ^b^
	C	0.02 ± 0.00 ^B^	0.03 ± 0.00 ^aA^	0.03 ± 0.00 ^A^	0.03 ± 0.00 ^aA^
Ʃ Amount of all aroma classes	A	2.33 ± 0.26 ^A^	2.17 ± 0.06 ^B^	1.83 ± 0.01 ^C^	1.42 ± 0.11 ^aD^
	B	2.30 ± 0.25 ^A^	2.03 ± 0.09 ^B^	1.47 ± 0.14 ^C^	1.28 ± 0.04 ^bD^
	C	2.61 ± 0.08 ^A^	2.07 ± 0.12 ^B^	1.38 ± 0.07 ^C^	1.05 ± 0.03 ^cD^

ND: Not detectable. ^A–D^ Means with different superscript in the same row significantly differ at *p* < 0.05. ^a–c^ Means with different superscript in the same column significantly differ at *p* < 0.05.

## Data Availability

The original contributions presented in the study are included in the article, further inquiries can be directed to the corresponding author.
